# Determinants of variability of five programmed death ligand-1 immunohistochemistry assays in non-small cell lung cancer samples

**DOI:** 10.18632/oncotarget.23827

**Published:** 2018-01-02

**Authors:** Ross A. Soo, Joey Sze Yun Lim, Bernadette Reyna Asuncion, Zul Fazreen, Maria Cynthia Herrera, Mohd Feroz Mohd Omar, Nguyen Hoang Diem Phuong, Ju Ee Seet, Benhur Amanuel, Barry Iacopetta, David Byrne, Shona Hendry, Stephen Fox, Richie Soong

**Affiliations:** ^1^ Cancer Science Institute of Singapore, National University of Singapore, Singapore, Singapore; ^2^ Department of Haematology-Oncology, National University Cancer Institute of Singapore, National University Health System, Singapore, Singapore; ^3^ School of Surgery, University of Western Australia, Perth, Australia; ^4^ Department of Pathology, National University Hospital, Singapore, Singapore; ^5^ Department of Anatomical Pathology, Queen Elizabeth II Medical Centre, Perth, Australia; ^6^ School of Pathology and Laboratory Medicine, The University of Western Australia, Perth, Australia; ^7^ Department of Pathology, Peter MacCallum Cancer Centre, Melbourne, Australia; ^8^ Department of Pathology, The University of Melbourne, Melbourne, Australia; ^9^ Department of Pathology, National University of Singapore, Singapore, Singapore

**Keywords:** programmed death ligand-1, immunohistochemistry, non-small cell lung cancer, immunotherapy

## Abstract

Programmed death ligand-1 (PD-L1) expression as determined by immunohistochemistry (IHC) is potentially predictive of clinical outcome. The aim of this study was to assess the concordance of reported PD-L1 IHC assays and investigate factors influencing variability. Consecutive sections from 20 non-small cell lung cancers (NSCLCs) comprising resection, core biopsy, cytology and pleural fluid samples underwent IHC with 5 different antibody/autostainer combinations: 22C3/Link48, 28-8/BOND-MAX, E1L3N/BOND-MAX, SP142/BenchMark and SP263/BenchMark. PD-L1 RNA levels were assessed using RNAscope. The frequency of positive cases using scoring thresholds from clinical trials was 72%, 33%, 61%, 56%, and 33% for the 5 IHC protocols respectively, and 33% for RNAscope. Pairwise agreement on the classification of cases as positive or negative for PD-L1 expression ranged from 61%-94%. On a continuous scale, the lowest correlation was between 28-8/BOND-MAX and SP142/BenchMark (R^2^=0.25) and highest was between 22C3/Link48 and E1L3N/BOND-MAX (R^2^=0.71). When cases were ordered according to tumor cell (TC)%, a similar ranking of cases across IHC protocols could be observed, albeit with different quanta and limits of detection. Single-slide OPAL 7-color fluorescence IHC analysis revealed a high degree of co-localization of staining from the 5 PD-L1 antibodies. Using SP142 antibody in a BOND-MAX protocol led to increased TC% quanta, while retaining a similar ranking of samples according to TC%. The results of this study highlight tumor PD-L1 status can vary significantly according to IHC protocol. Protocol-dependent staining intensities and nominated thresholds for positivity contribute to this variability, while the antibody used appears to be less of a factor.

## INTRODUCTION

In patients with advanced non-small cell lung cancer (NSCLC) treated with programmed death-1 (PD-1)/programmed death ligand-1 (PD-L1) inhibitors, survival benefit is seen in only a subset of patients [1, 2]. Accumulating evidence suggests that PD-L1 expression determined by immunohistochemistry (IHC) may be a useful biomarker for identifying patients who might benefit from PD-1/PD-L1 inhibitors [3]. However, different assays to assess PD-L1 expression have been used for different PD-1/PD-L1 inhibitors [4]. To select for pembrolizumab, the recommended companion *in vitro* diagnostic (IVD) assay is the 22C3 antibody (Ab) clone with the Dako Link 48 autostainer [5, 6]. For nivolumab, it is the 28-8 clone with the Dako Link 48 autostainer [7], while for durvalumab it is the SP263 clone with the Ventana BenchMark Ultra autostainer [8, 9]. For atezolizumab, tumor and immune cell PD-L1 expression are assessed using the Ab clone SP142 with the Ventana BenchMark Ultra autostainer [10, 11]. In-house or laboratory-developed tests (LDTs) for PD-L1 expression have also been used in the clinical setting [12]. The different IHC Ab clones, staining protocols, staining platforms, scoring systems and thresholds for positivity have led to considerable complexity in the assessment of PD-L1 expression [4]. For most diagnostic laboratories, it is neither feasible nor cost-effective to provide the full range of PD-L1 assays. There is clearly a need to reduce the complexity of PD-L1 testing and to make it more robust and accessible. The aims of this study were therefore to systematically compare the results from commercially available PD-L1 IHC assays in clinically relevant NSCLC samples, and identify factors affecting the classification of PD-L1 status.

## RESULTS

### PD-L1 IHC staining in tumor and immune cells

**Figure 1:**
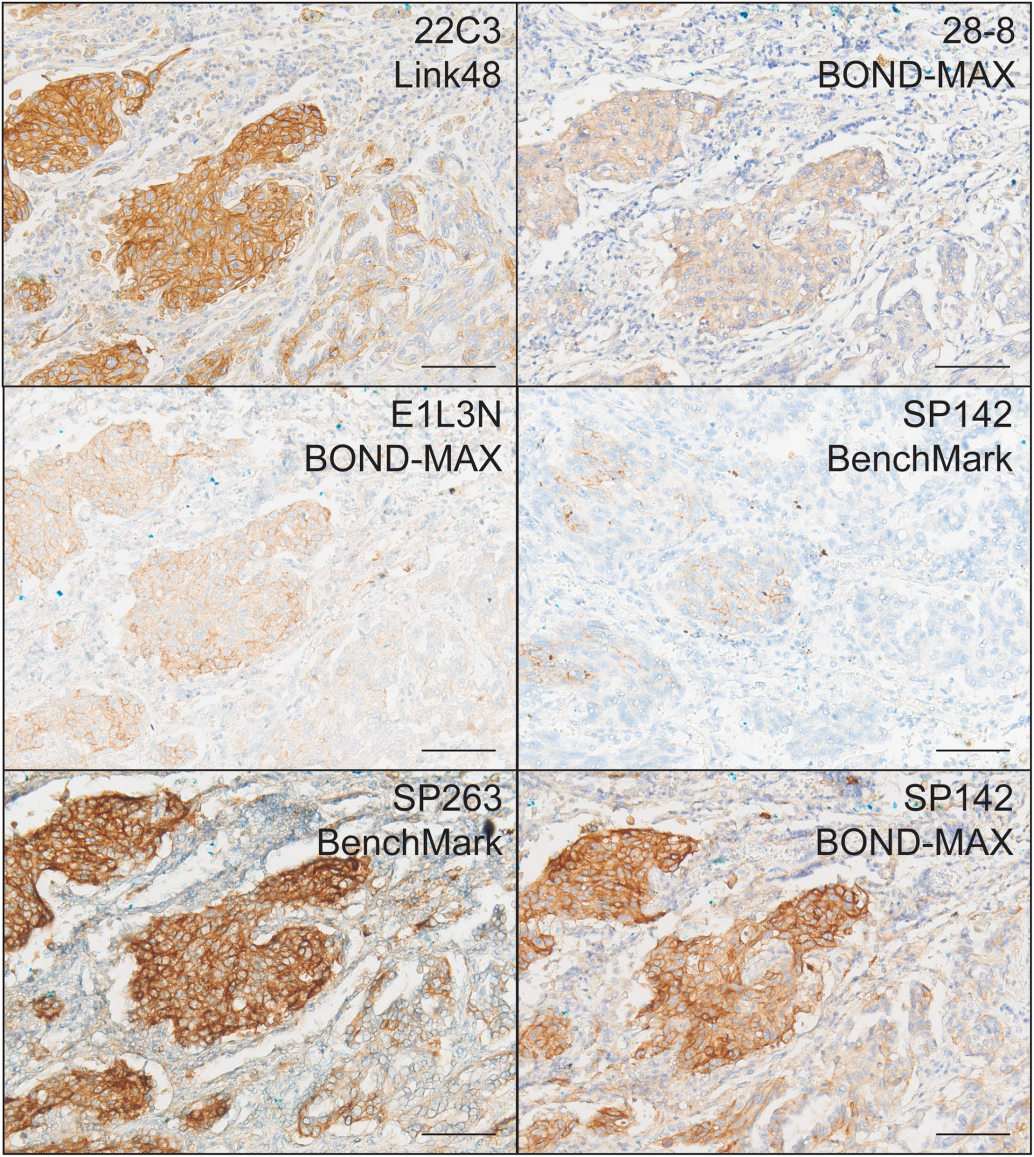
Representative IHC stains of PD-L1 in NSCLC using 22C3/Link48, 28-8/BOND-MAX, E1L3N/BOND-MAX, SP142/BenchMark, SP263/BenchMark, and SP142 BOND-MAX protocols Scale bar = 150 μm.

Application of the recommended IHC protocols (Supplementary Table 1) for the 5 PD-L1 Abs resulted in specific staining of epithelial cells in placental tissue (Supplementary Figure 1). In NSCLC samples, staining was predominantly localized to TCs, with occasional faint blushes in the stroma (Figure 1). Staining of TCs was observed primarily at the membrane, as well as in the cytoplasm of some cases at lower intensity. There were notable differences in staining intensity between protocols, with staining observed to be strongest with SP263/Benchmark, moderate with 22C3/Link48, 28-8/BOND-MAX and E1L3N/BOND-MAX, and weak with SP142/BenchMark.

Staining of tumor-infiltrating ICs was not assessable in cell blocks and fine needle aspirates (FNAs) due to the nature of the samples. In resection and small biopsy samples, staining was observed in occasional aggregates of lymphocytes and neutrophils using all 5 protocols, and more noticeably with SP142/BenchMark, 22C3/Link48 and 28-8/BOND-MAX. Staining was also observed in islands of macrophages, which were relatively abundant in most samples. Given the small sample size after exclusion of cell blocks and FNAs, comparison of IC PD-L1 expression between assays was not performed.

### Comparison of PD-L1 assay results

**Figure 2:**
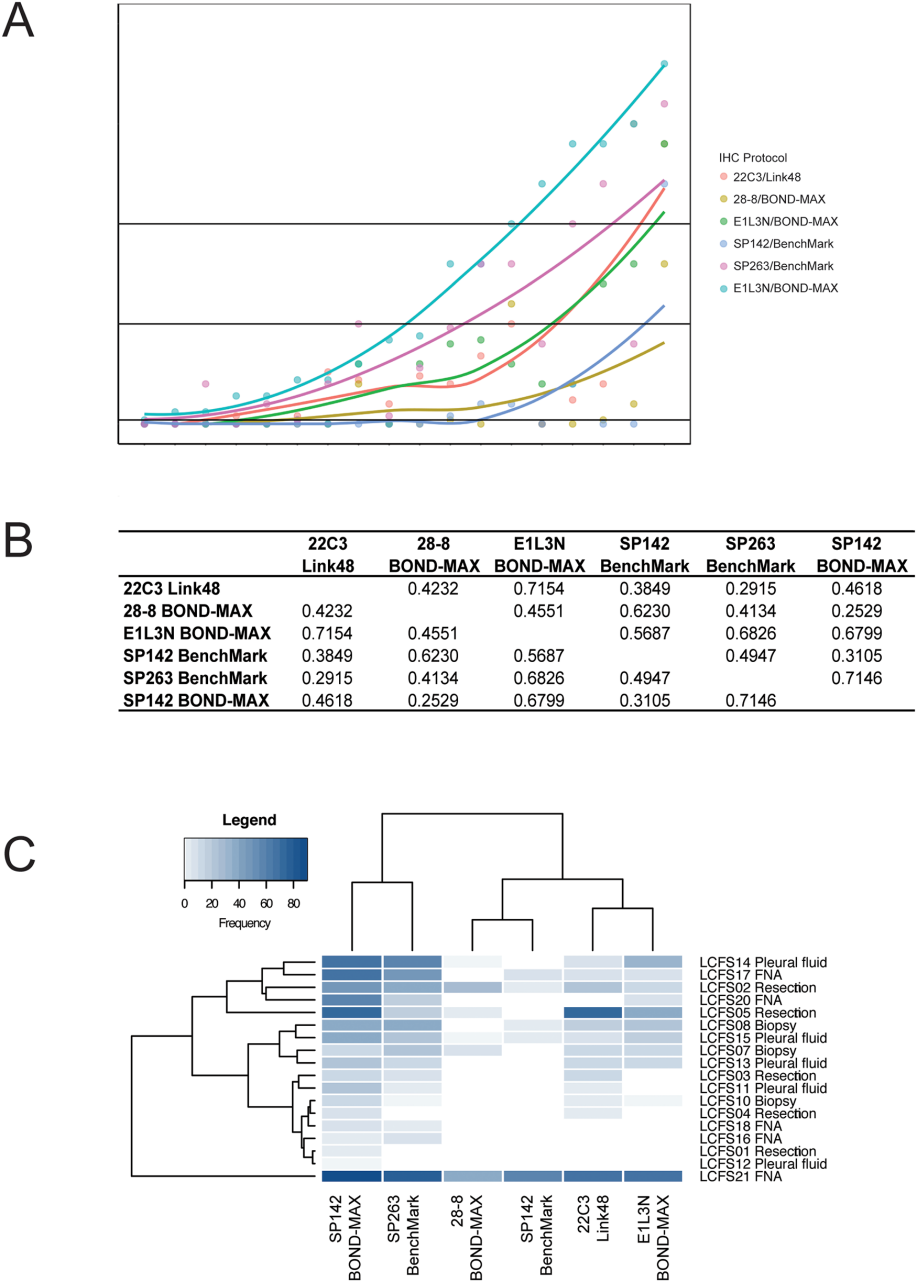
**(A)** Cumulative frequencies of cases considered IHC positive according to the IHC protocols tested in this study. Cases are sorted in order of increasing TC% using SP263/BenchMark. **(B)** Pairwise correlation between the IHC protocols in TC% as a continuous variable. The R^2^ value from Spearman's rank test is indicated. **(C)** Unsupervised hierarchical clustering of TC% scores according to IHC protocol and cases. The sample type of each case is indicated to the right of the heatmap.

The distribution of TC% staining in each sample according to PD-L1 IHC protocol is shown in Figure 2A. Table 1 summarizes the frequency of positive cases according to the various IHC protocols, Abs and scoring thresholds. Using the recommended IHC protocols and the scoring thresholds reported in clinical trials, the frequency of positive cases was 72%, 33%, 61%, 56%, and 33% for 22C3/Link48, 28-8/BOND-MAX, E1L3N/BOND-MAX, SP142/BenchMark, and SP263/BenchMark, respectively. Table 2 shows the pairwise agreement between clinically relevant protocols for the classification of PD-L1 expression, ranging from 22% (SP142/Benchmark TC50/TC10 and SP263/Benchmark TC1) to 94% (22C3/Link48 TC50 and SP142/BenchMark TC50/IC10).

**Table 1:** Frequency of positive cases according to different IHC protocols and scoring thresholds

Protocol	TC1	TC1/IC1	TC25	TC50	TC50/IC10
22C3 Link48 (IVD)	13 (72%)	14 (78%)	3 (17%)	2 (11%)	3 (17%)
28-8 BOND-MAX	6 (33%)	11 (61%)	2 (11%)	0 (0%)	1 (6%)
E1L3N BOND-MAX	11 (61%)	14 (78%)	3 (17%)	1 (6%)	2 (11%)
SP142 BenchMark (IVD)	5 (28%)	10 (56%)	1 (6%)	1 (6%)	1 (6%)
SP263 BenchMark (IVD)	15 (83%)	17 (94%)	6 (33%)	3 (17%)	4 (22%)
SP142 BOND-MAX	18 (100%)	18 (100%)	8 (44%)	6 (33%)	9 (50%)

IC, immune cells; IVD, *in-vitro* diagnostic; TC, tumor cell; The number following TC or IC indicates the threshold of percentage for considering a case as positive. “/” indicates “or”.

**Table 2:** Concordance in cases considered IHC positive and IHC negative according to published IHC and antibody protocols

	22C3/Link48 TC50			28-8/BOND-MAX TC1			SP142/BenchMark TC1/IC1			SP142/BenchMark TC50/IC10			SP263/BenchMark TC1			SP263/BenchMark TC25		
	-/−	+/+	Conc.	-/−	+/+	Conc.	-/−	+/+	Conc.	-/−	+/+	Conc.	-/−	+/+	Conc.	-/−	+/+	Conc.
22C3/Link48 TC1	5	2	7 (39%)	5	6	11 (61%)	5	10	15 (83%)	5	1	6 (33%)	2	12	14 (78%)	5	6	11 (61%)
22C3/Link48 TC50				12	2	14 (78%)	8	2	10 (56%)	16	1	17 (94%)	3	2	5 (28%)	11	1	12 (67%)
28-8/BOND-MAX TC1							7	5	12 (57%)	12	1	13 (72%)	3	6	9 (50%)	10	4	14 (78%)
SP142/BenchMark TC1/IC1										8	1	9 (50%)	2	9	11 (61%)	7	5	12 (67%)
SP142/BenchMark TC50/IC10													3	1	4 (22%)	12	1	13 (72%)
SP263/BenchMark TC1																3	6	9 (50%)

IC, immune cells; TC, tumor cell; The number following TC or IC indicates the threshold of percentage for considering a case as positive. “/” in dicates “or”. -/− = amount of cases negative for both protocols, +/+ amount of cases positive for both protocols; Conc. = concordance, of 18 cases.

Considerable variation in the pairwise correlation of TC% was observed for different protocols, with the lowest between 28-8/BOND-MAX and SP142/BenchMark (R^2^=0.25) and the highest between 22C3/Link48 and E1L3N/BOND-MAX (R^2^=0.71) (Figure 2). Unsupervised hierarchical clustering of TC% values revealed three subgroups: 22C3/Link48 and E1L3N/BOND-MAX, 28-8/BOND-MAX and SP142/BenchMark, and SP263/BenchMark alone. There was no obvious clustering of cases according to the sample type, indicating that PD-L1 TC% scoring was not biased by this factor. Similar trends in the ranking of cases by TC% were observed across IHC protocols. Cases with the highest or lowest TC% were generally the same across different IHC protocols. Cases with low TC% in protocols that were generally associated with strong staining (i.e. protocols using SP263/BenchMark and 22C3/Link48) often showed no detectable staining with protocols associated with weaker staining (i.e. protocols using SP142/BenchMark, 28-8/BOND-MAX and E1L3N/BOND-MAX). This suggests the IHC protocols stained cases in mostly the same rank, but differed with respect to the staining intensity and detection limit, consistent with a concentration effect.

### Correlation between IHC and RNA *in-situ* hybridization for PD-L1 expression

To provide a reference for PD-L1 expression independent of the IHC protocol, PD-L1 RNA expression was assessed by RNA *in-situ* hybridization using RNAscope. Staining was visible as brown dots or clusters predominantly in tumor and occasionally in the mesenchyme (Supplementary Figure 2). Positive PD-L1 RNA expression was seen in 6 of 18 (33%) samples. The sensitivity and specificity of each IHC assay at various thresholds is summarized in Supplementary Table 2. Only the results from the E1L3N/BOND-MAX IHC assay correlated significantly with positive RNA expression (*p*=0.038).

### Co-localization of staining according to multimarker fluorescence IHC

**Figure 3:**
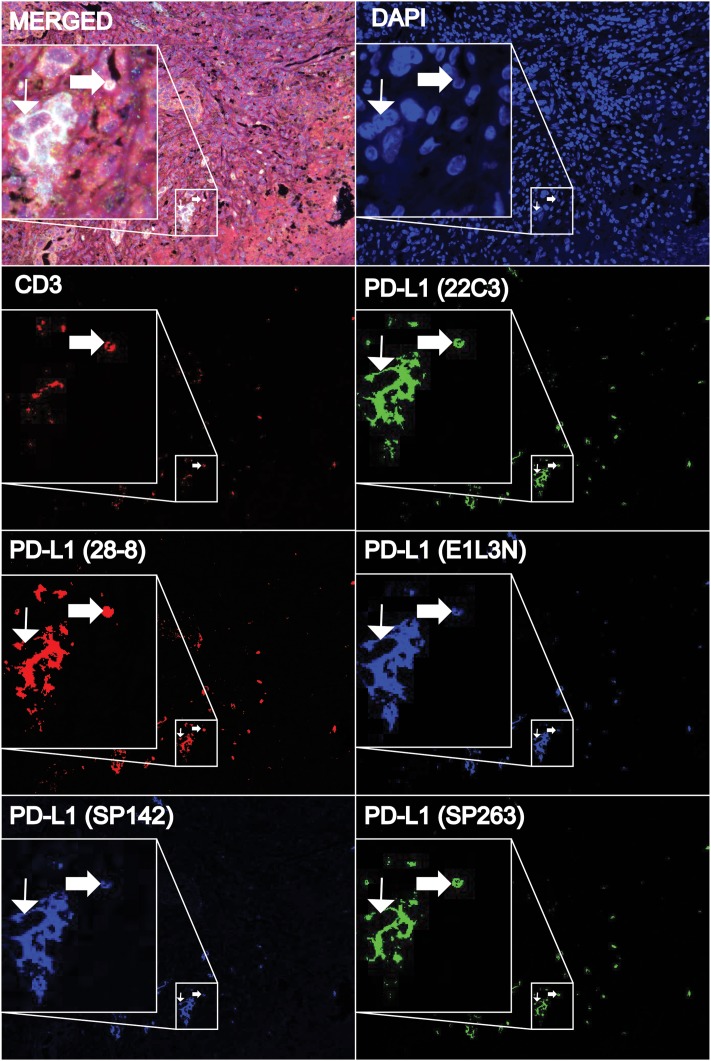
Representative multimarker fluorescence staining of NSCLC tissue samples using DAPI, CD3, and five PD-L1 antibodies (22C3, 28-8, E1L3N, SP142, and SP263) to assess their co-localization A high degree of co-localization of staining amongst all PD-L1 antibodies in tumor (thin arrow) and CD3+ immune cells (thick arrow) can be seen in the merged image, together with the individual unmixed images. Each inset displays a magnified focus of the 20x image.

Differences in staining results between protocols may have been due to the different affinities of each Ab for TCs. To study the binding of multiple Abs to the same cells, OPAL 7-plex fluorescence IHC staining was performed using the 5 PD-L1 Abs, a CD3 Ab and DAPI on a single tissue section. An optimal staining protocol was determined through testing titrations of individual Abs, testing the staining of each Ab with each fluorophore as a single stain, testing different antigen retrieval conditions, and confirming there were no difference in staining patterns from changing the order of application of the Abs in multiplex staining (results not shown). Using the optimized protocol, co-localized staining of all 5 PD-L1 Abs was seen in the outer epithelial layer of the placenta (Supplementary Figure 3). TC PD-L1 staining was observed in 6 of 18 (33%) NSCLC samples (Figure 3). In all cases, a high degree of co-localization for PD-L1 Ab staining was observed in both tumor and CD3-positive cells. The lesser degree of co-localization of CD3 staining with PD-L1 staining in placenta and NSCLC tissue supported the specificity of co-localized PD-L1 staining.

### Effect of an alternative IHC protocol using the same Ab

To test the influence of IHC protocol on apparent PD-L1 expression, an alternative IHC protocol was used for the Ab associated with the weakest staining, SP142. The “SP142/BOND-MAX” IHC protocol gave rise to a higher intensity of staining compared to the original SP142/BenchMark IHC protocol (Figures 1 and 2), thus demonstrating how the IHC protocol can influence the apparent level of PD-L1 expression. Results from the alternative SP142/BOND-MAX protocol showed the strongest correlation with the SP263/BenchMark protocol (R^2^=0.71), rather than the 28-8/BOND-MAX protocol observed previously (R^2^=0.62 with SP142/BenchMark). This was likely due to the stronger staining observed with the new SP142/BOND-MAX protocol.

## DISCUSSION

Recently, a number of results on the comparability of PD-L1 assays were reported. The Blueprint study compared validated clinical trial assays using the 22C3, 28-8, SP142 and SP263 Abs on 39 resected and biopsy NSCLC samples [13]. The IHC assays using 28-8, 22C3, and SP263 gave similar results for TC staining, while all four assays also detected IC staining, albeit with greater variation. Misclassification of PD-L1 status was observed when applying thresholds from other assays. Another study compared IVD assays (using 22C3 and 28-8) and LDTs (using E1L3N and SP142) in 90 resected NSCLC samples evaluated by 13 pathologists. The investigators observed similar TC staining for the 22C3, 28-8 and E1L3N assays, with results from the latter two showing significant correlation [14]. Consistent with the Blueprint study, weaker staining was seen with the SP142 assay, while IC staining was also less consistent. Ratcliffe *et al*. compared three assays using 22C3, 28-8 and SP263 on 493 resected NSCLC samples from clinical trials. They found a high degree of concordance for TC staining between the three assays using multiple scoring thresholds [15]. Neuman and colleagues reported a strong correlation between results from an IVD assay comprising 22C3 and the Dako Link 48 autostainer, and an LDT using 22C3 and the Ventana BenchMark XT autostainer [12], thus supporting the validity of the latter assay. More recently, Adam *et al.* evaluated PD-L1 expression in 41 resected NSCLC samples in a large, multi-centre French study aimed at harmonizing protocols. They reported that 14 of 27 (52%) LDTs using 22C3, 28-8 or SP263 gave comparable TC staining, but not IC staining, compared to the corresponding IVD assays [16].

Although the goals of the current study were similar to those of the recent reports, the current results nonetheless still have an important role in conveying additional independent experience from PD-L1 staining from the Asia-Pacific region. This experience is pertinent given that PD-L1 testing will ultimately be performed by many smaller, routine clinical laboratories around the world. Consistent with the above reports, relatively strong staining with the IVD assays involving 22C3/Link48 and SP263/BenchMark, and weak staining with the SP142/BenchMark assay was observed (Figures 1 and 2). In contrast with previous reports, staining with 28-8/BOND-MAX correlated more with that of SP142/BenchMark than with 22C3/Link48 or SP263/BenchMark (Figure 2). This may be due to the use of 28-8 on the BOND-MAX autostainer in this study, as it was the protocol available at study initiation. Another factor may have been the small sample size of this study, which was limited to make feasible the additional investigations performed. Nonetheless, the overall conclusion of the comparison in the current study ultimately reverberates other reports: the current heterogeneity in staining protocols and scoring thresholds can lead to significant variability in the classification of PD-L1 status of cases (Tables 1 and 2), and approaches to reduce the variability in classification are urgently needed.

Beyond the comparison, a key feature of this study was the additional observation that a “concentration effect” could be a basis for differences in results (Figure 2). The implication is the observed differences in results may not be due to the differing specificity of PD-L1 Abs to different proteins and resultant labelling of cells, but rather from the nominated IHC protocols and Ab concentrations of the assays. This is an important distinction, as the latter prospect opens the possibility of harmonization through protocol adjustment, while differential protein binding presents a challenge to harmonization. With these considerations, the observation of a high concordance in the localization of staining from the five PD-L1 Ab clones through a single-slide, 7-plex fluorescence staining (Figure 3) becomes an important achievement of this study. This is supported by the demonstration that simply changing a staining protocol can convert an apparently weak intensity staining Ab (SP142) into one with strong intensity staining (Table 1, Figure 2), highlighting the greater influence of protocol than Ab on perceived PD-L1 expression.

**Figure 4:**
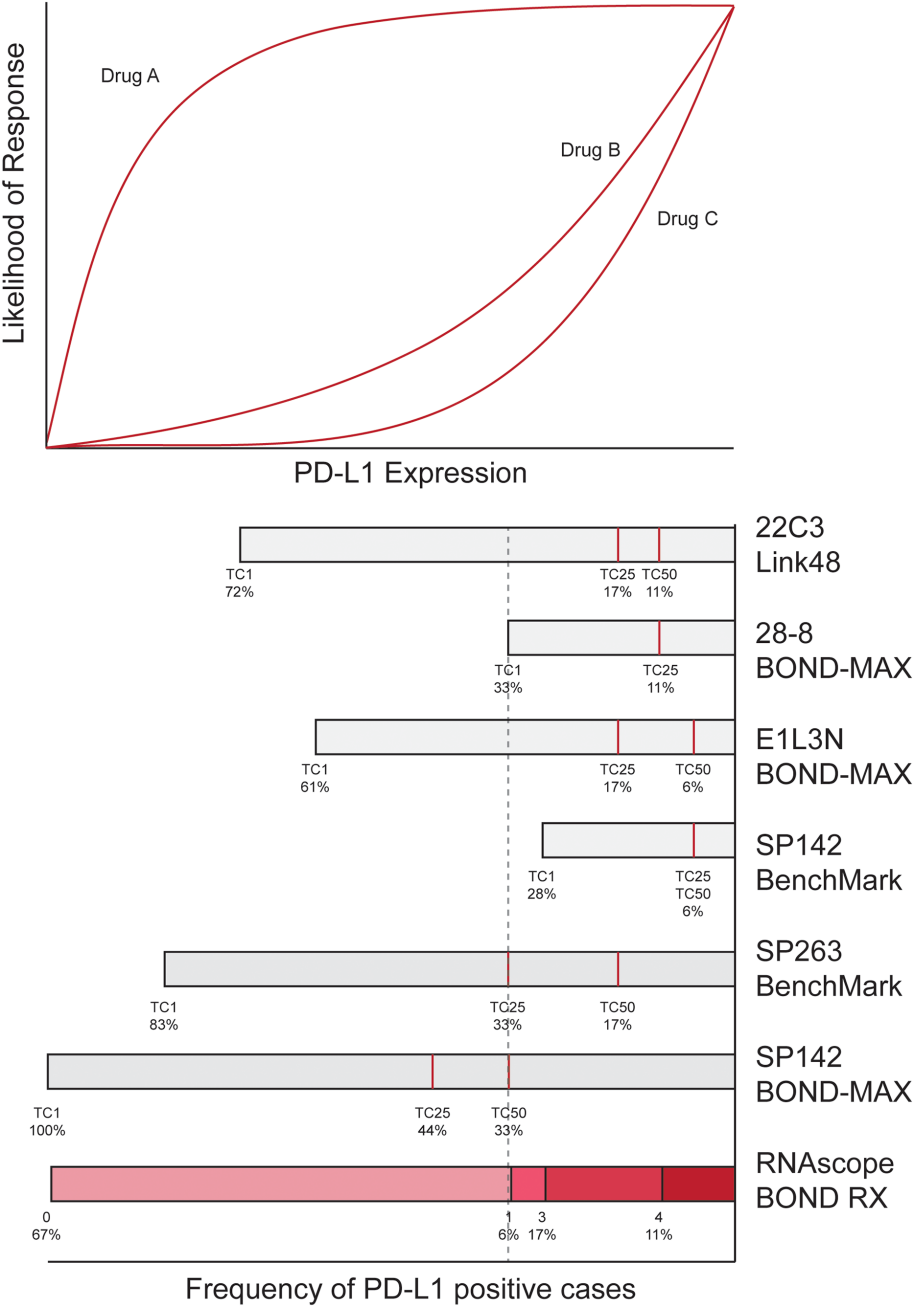
Model for conceptualizing the relationship between results from different assays and scoring thresholds, and the continuum of case volume and likelihood of response The chart at the top depicts three theoretical likelihood of response dynamics (hypothetical drugs A, B or C) according to PD-L1 expression, based on the premise that the likelihood of response increases with higher PD-L1 expression in a non-linear fashion. Aligned below the chart is a PD-L1 assay scoring conversion model. This model was created by drawing to scale the length of bars from the right-hand side according to the frequency of PD-L1 positive cases observed for each respective PD-L1 antibody (Table 1). Red lines inside the bars denote the frequency observed at respective scoring thresholds. Aligned in this way, the relationship between assay dynamic ranges, scoring thresholds, case volume and likelihood of benefit can be conceptualized. For example, it can be seen that a TC25 threshold using the SP263/BenchMark assay aligns to TC1 using 22C3/Link48 and 28-8/BOND-MAX assays, between TC1 to TC25 using E1L3N/BOND-MAX, TC1 using SP142/BenchMark, TC50 using SP142/BOND-MAX and a RNAscope/BOND RX score of one (dashed line).

The results of this study provide a framework for understanding the relationships between the dynamic ranges of different PD-L1 assays, the scoring thresholds, and the likelihood of response to treatment (Figure 4). The evidence for a concentration effect supports aligning the dynamic range of each assay according to the frequency of positive cases at the respective thresholds (Table 1). Through this alignment, the observed dynamic ranges, relationships between respective thresholds, and reasons for the variable classification of samples according to the assays and thresholds used can be better conceptualized. It is interesting to note the alignment of many independently nominated thresholds used for clinical trials and publications, namely 28-8/BOND-MAX TC1, SP142/BenchMark TC1, SP263/BenchMark TC25 and RNAscope/BOND RX Score 1. This is perhaps due to the intrinsic relevance of the biological PD-L1 expression level. For added value, theoretical dose-response curves are superimposed above the dynamic ranges, thereby helping to visualize optimal protocol points for maximizing response rates and case volumes along the continua of response likelihood. This model awaits further update with more advanced data as it becomes available, but nevertheless is a guide for how future information can be reconciled and visualized.

In conclusion, PD-L1 testing is currently at a crossroad. On one hand, many studies have reported the value of PD-L1 testing as a predictor for response to PD-L1/PD-1 therapy, and regulatory approval has been obtained for some assays [17]. However, the present study and others [4, 18] reveal concerns about the current state of PD-L1 testing, in particular the irregularities in classification, and the confusion and inefficiencies arising from the many different assay options [19, 20]. In this context, the distinction provided by this study that staining protocol contributed more to variability than biology (e.g. antibody affinity) should help give confidence that protocol harmonization is achievable. The conceptual model presented for reconciling dynamic ranges, thresholds and response continua (Figure 4) should also help to reduce confusion. Standardization of PD-L1 testing is eagerly awaited, including by those studying other emerging biomarkers for PD-L1/PD-1 inhibitors such as mutation load, neo-antigen load, and tumor infiltrating lymphocytes [19, 21]. Future work should reveal whether these features can be integrated with PD-L1 expression to improve treatment selection. More work is also needed to assess the impact of inter-observer and inter-laboratory variability, pre-analytical preparation, and the potential of digital slide imaging.

## MATERIALS AND METHODS

### Samples

Twenty formalin-fixed and paraffin-embedded (FFPE) tissue blocks, comprising 5 resections, 5 core or bronchial biopsies, 5 pleural fluid cell blocks and 5 fine needle aspiration (FNA) cell blocks from 20 different NSCLC patients were obtained by a study pathologist (JES). Ten consecutive full tissue sections of 4μm thickness were prepared from these blocks. One section from each series underwent standard hematoxylin and eosin staining and was reviewed by a study pathologist (BRA) to ensure 100 tumor cells of sufficient quality for assessment were present. One FNA cell block sample had inadequate tumor and was replaced by an alternative FNA sample from a new case. During processing, the staining for two bronchial core biopsies did not pass quality control for all five Abs and were therefore not considered further, leaving 18 samples in the overall analysis. After sectioning, the slides were stored at −20°C and used within 6 months. All samples were obtained according to institutionally approved protocols.

### Immunohistochemistry (IHC)

IHC was performed according to IVD protocols for the 22C3 Ab on the Dako Autostainer Link 48 (Agilent, Santa Clara, CA), and the SP142 and SP263 clones on the Ventana BenchMark XT (Roche Ventana, Tuscon, AZ) (Supplementary Table 1). The remaining assays were conducted as LDTs. IHC using the 28-8 clone (concentration 1:500; Abcam, Cambridge, UK) and E1L3N (1:200; Cell Signaling Technologies, Beverly, MA) was performed on the BOND-MAX autostainer (Leica, Wetzlar, Germany). Positive and negative controls were included and consisted of placental tissue and sections stained without primary Ab, respectively.

IHC stains were independently scored by three pathologists (BRA, MCH, SH), according to vendors’ recommendations. Tumor staining was scored according to the extent of cell staining (0%, 1%, 5%, 10%, and then nearest 5% from 11-100%) at each level of staining intensity from 0-3 (0, none; 1, partial; 2, compete and faint; 3 complete and strong). IC were scored as previously described [10, 22], specifically as IC0 (<1% of IC staining at any intensity), IC1 (1-4%), IC2 (5-9%), and IC3 (≥10%). Raw scores were then converted to comply with vendor specifications. To facilitate analysis, the scores from a single pathologist (BRA) were used, following confirmation of an adequate agreement in scores between pathologists (Cohen's kappa test, p<0.05).

### Multimarker fluorescence IHC

Assessment of staining co-localization was performed using the OPAL 7-color fIHC Kit (Perkin Elmer, Waltham, MA) and the BOND RX autostainer (Leica). Following dewax and rehydration, samples underwent antigen retrieval at pH6 for 20 mins, and diluent was used for protein blocking. The concentration and fluorophores for the Abs were: 22C3 (1:500, Opal570), 28-8 (1:1500, Opal650), SP142 (1:2000, Opal520), SP263 (1:5, Opal620), E1L3N (1:2000, Opal540) and CD3 (1:1000, Opal690). Each Ab was incubated for 15 mins at room temperature, and detected using the Bond Polymer Refine Detection Kit (Leica). Slides were mounted with VECTASHIELD Mounting Medium with DAPI (Perkin Elmer) and imaged using the Vectra Slide Analysis System V2.0 (Perkin Elmer).

### RNA *in-situ* staining

RNA *in-situ* staining was performed with the RNAscope 2.5 LS (Brown) Reagent Kit (Advanced Cell Diagnostics, Newark, CA) and the BOND RX autostainer (Leica). Samples were incubated with ACD HIER for 15 mins at 95°C, then pre-treated using ACD Protease III at 40°C for 15 mins, then incubated with RTU PD-L1 RNA probes at 40°C for 120 mins. ACD AMP 1-6 was applied at 40°C for 15-30 mins for signal amplification before application of BOND Polymer Refine Detection kit (Leica). Samples were also stained with PPIB probes at 40°C for 120 mins as positive controls. Stains were scored according to vendor recommendations on dots within a cell boundary: 0 (0-1 dots per 10 cells at 40x magnification), 1 (1-3 dots per cell visible at 20–40x magnification), 2 (4-10 dots per cell or very few dot clusters visible at 20–40x magnification), 3 (>10 dots per cell, with <10% of cells with densely clustered dots at 20x magnification) and 4 (>10 dots per cell, with ≥10% of cells with densely clustered dots at 20x magnification). A score of 1 or more was considered as PD-L1 positive.

### Statistical analysis

Pairwise associations between assays was assessed using Fisher's exact test for categorical variables and Spearman's rank test for continuous variables. Sensitivity was calculated as true positives / (true positives + false negatives) x 100, and specificity as true negatives / (false positives + true negatives) x 100, where these classifications were based on RNA results. Unsupervised hierarchical clustering analysis was performed using RStudio, based on Euclidean and Spearman methods [23]. Significance was considered as p<0.05.

## SUPPLEMENTARY MATERIALS FIGURES AND TABLES



## Abbreviations

Ab: antibody
fIHC: fluorescence immunohistochemistry
FNA: fine needle aspirate
IC: immune cell
IHC: immunohistochemistry
IVD: *in-vitro* diagnostic
LDT: laboratory developed test
NSCLC: non-small cell lung cancer
PD-1: programmed death-1
PD-L1: programmed death ligand-1
TC: tumour cell.
