# Supplementary material for: Determinants of variability of five programmed death ligand-1 immunohistochemistry assays in non-small cell lung cancer samples
Source: Oncotarget. 2018 Jan 2;9(6):6841–51. doi: 10.18632/oncotarget.23827 (PMC5805519; doi:10.18632/oncotarget.23827)
Supplement: Supplementary file 1 [file oncotarget-09-6841-s001.pdf]

Determinants of variability of five programmed death ligand-1 immunohistochemistry assays in non-small cell lung cancer samples

SUPPLEMENTARY MERATALS

REFERENCES

1. Herbst RS, Baas P, Kim DW, Felip E, Pérez-Gracia JL, Han JY, Molina J, Kim JH, Arvis CD, Ahn MJ, Majem M, Fidler MJ, de Castro G Jr, et al. Pembrolizumab versus docetaxel for previously treated, PD-L1-positive, advanced non-small-cell lung cancer (KEYNOTE-10): a randomized controlled trial. Lancet. 2016; 387: 1540–50.
2. Borghaei H, Paz-Ares L, Horn L, Spigel DR, Steins M, Ready NE, Chow LQ, Vokes EE, Felip E, Holgado E, Barlesi F, Kohlhäufl M, Arrieta O, et al. Nivolumab versus docetaxel in advanced nonsquamous non-small-cell lung cancer. N Engl J Med. 2015; 373: 1627–39.
3. http://www.ventana.com/ventana-pd-l1-sp263-rabbit-monoclonal-primary-antibody-2/ (accessed April 17th 2017.)
4. https://www.cellsignal.com/products/primary-antibodies/pd-l1-e1l3n-xp-rabbit-mab/13684 (accessed April 17th 2017).
5. Rittmeyer A, Barlesi F, Waterkamp D, Park K, Ciardiello F, von Pawel J, Gadgeel SM, Hida T, Kowalski DM, Dols MC, Cortinovis DL, Leach J, Polikoff J, et al. Atezolizumab versus docetaxel in patients with previously treated non-small-cell lung cancer (OAK): a phase 3, open-label, multicentre randomised controlled trial. Lancet. 2017; 389: 255–65.
6. http://productlibrary.ventana.com/ventana_portal/OpenOverlayServlet?launchIndex=1&objectId=741-49051014258EN (accessed April 17th 2017).
7. Garassino M, Vansteenkiste JF, Kim J, Léna H, Mazières J, Powderly J, Dennis P, Huang Y, Wadsworth C, Rizvi N. Durvalumab in ≥3rd-line locally advanced or metastatic, EGFR/ALK wild-type NSCLC: results from the phase 2 ATLANTIC study. J Thorac Oncol. 2017; 12: S10–11.

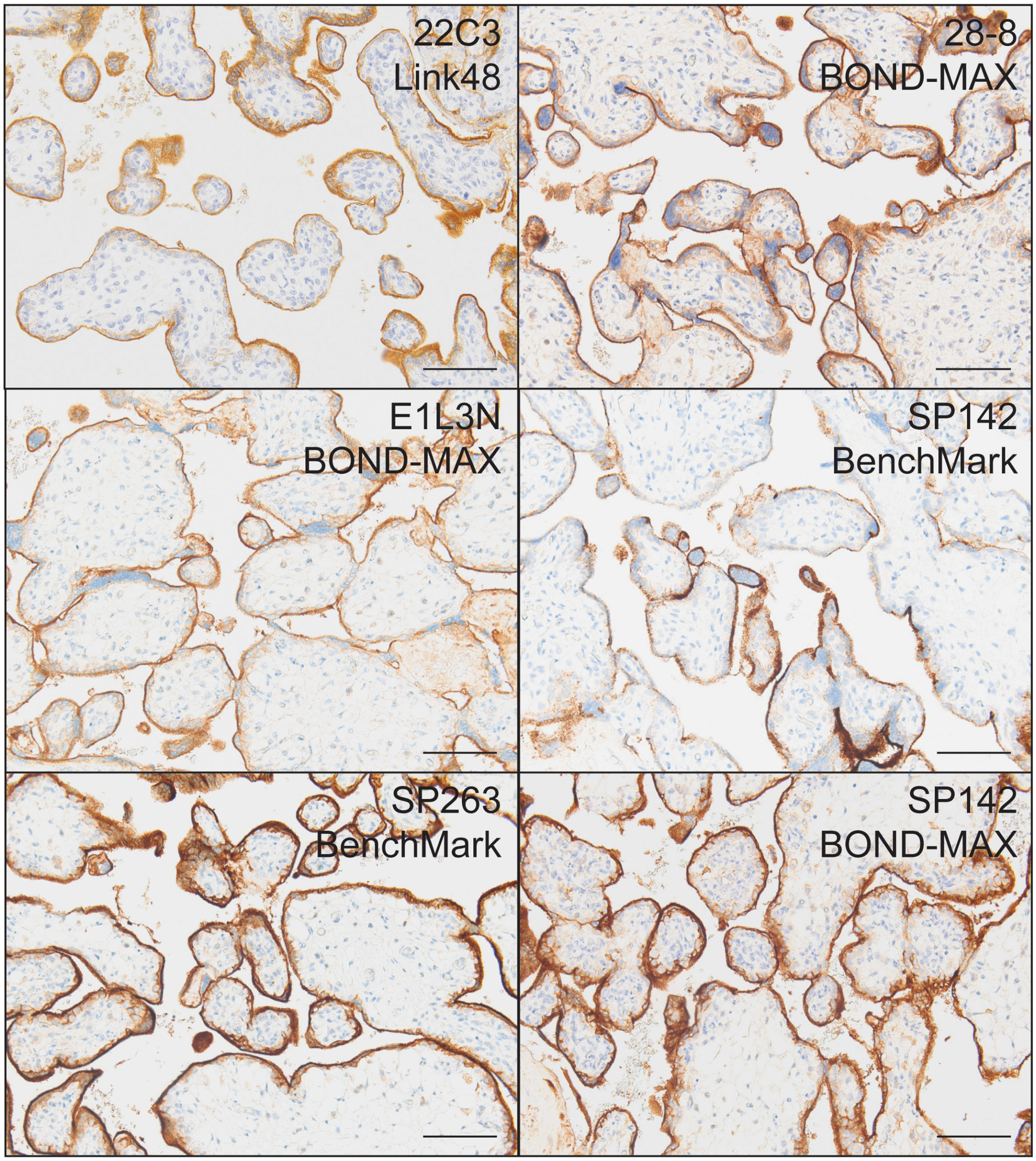

Supplementary Figure 1: Representative immunohistochemistry stains of PD-L1 expression in human placenta with 22C3/Link48, 28-8/BOND-MAX, E1L3N/BOND-MAX, SP142/BenchMark, and SP263/BenchMark protocols. Staining was specifically observed in the trophoblast layer but not in stromal components. 20x magnification.

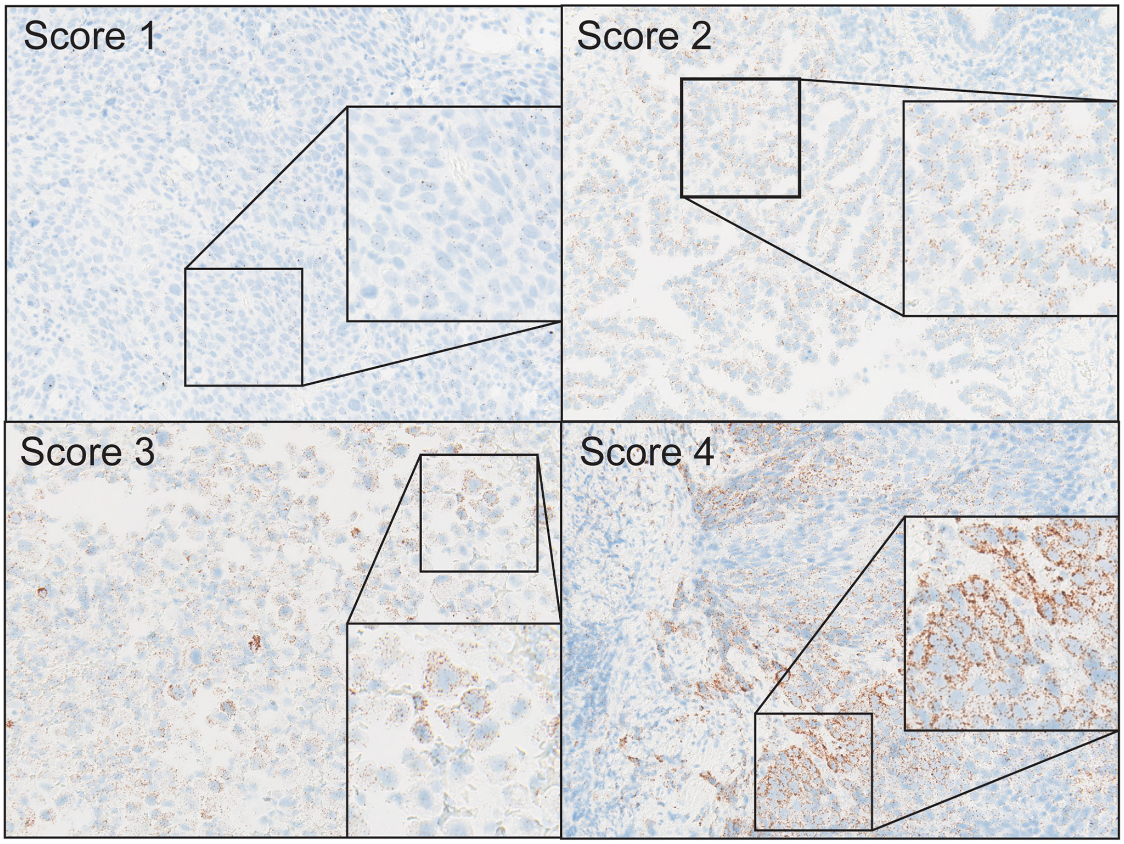

Supplementary Figure 2: PD-L1 mRNA signals using RNAscope displaying different degree of staining according to score. Each inset displays a magnified focus of the 20x image.

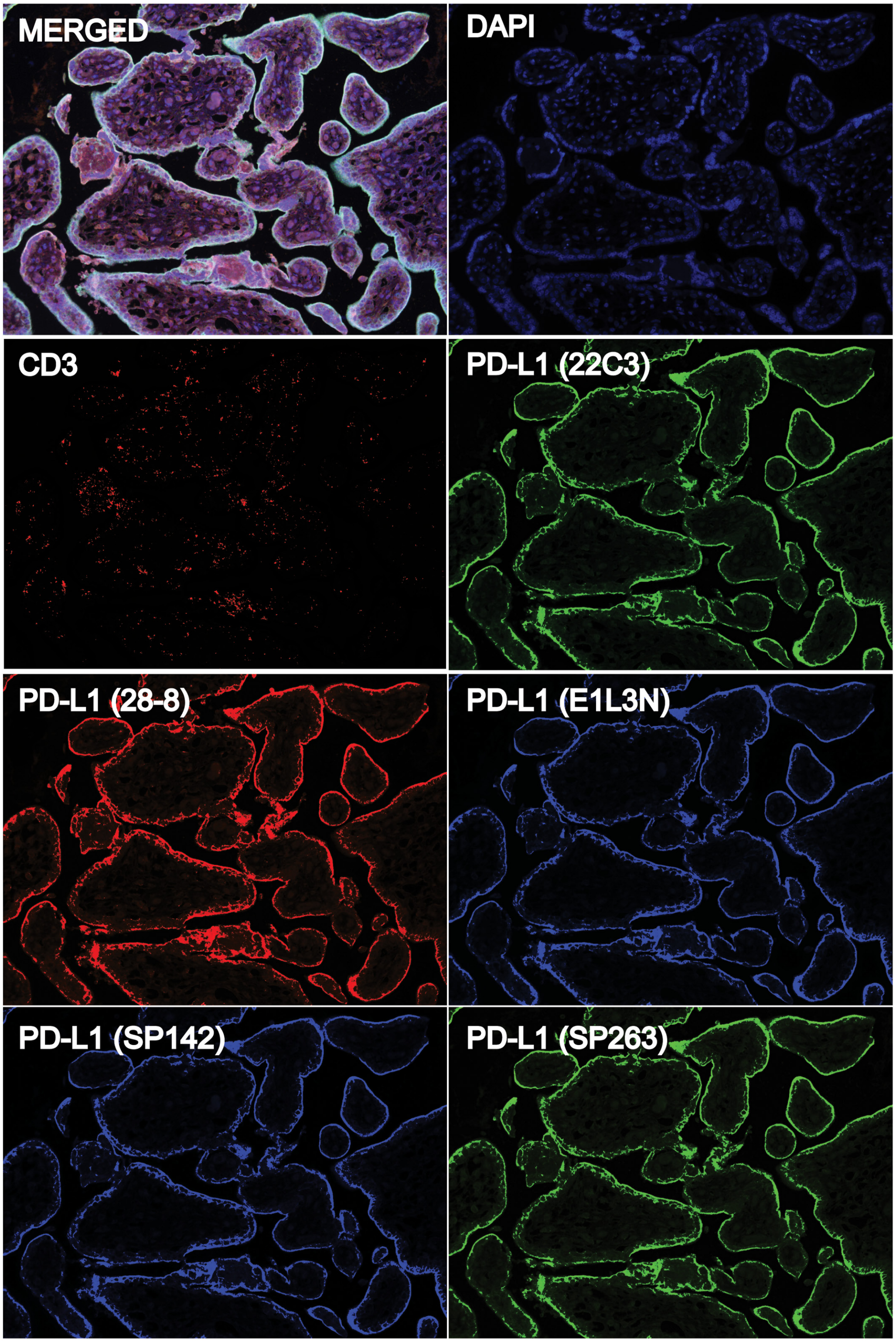

Supplementary Figure 3: Representative fluorescence multimarker staining of placenta tissue using DAPI, CD3, five PD-L1 antibodies (22C3, 28-8, E1L3N, SP142, and SP263) to assess for co-localization. 20x magnification.

Supplementary Table 1: Details of the PD-L1 assays used in the current study

	22C3/Link48 [1]	28-8/BOND-MAX [2]	E1L3N/ BOND-MAX [4]	SP142/ BenchMark [5]	SP263/ BenchMark [3]	SP142/BOND-MAX
Assay Type	*in-vitro* diagnostic	lab-developed test	lab-developed test	lab-developed test	*in-vitro* diagnostic	lab-developed test
Primary antibody clone	22C3, mouse monoclonal	28-8, rabbit monoclonal	E1L3N, rabbit monoclonal	SP142, rabbit monoclonal	SP263, rabbit monoclonal	SP142, rabbit monoclonal
Source	Dako, Carpinteria, CA	Abcam, Cambridge, UK	Cell Signaling Technologies, Beverly, MA	Roche Diagnostics, Mannheim, Germany	Roche Ventana, Tuscon, AZ	Roche Diagnostics, Mannheim, Germany
Concentration	Ready-to-Use (3µg/mL)	1:500 (2µg/mL)	1:200 (5.05µg/ mL)	Ready-to-Use (7µg/mL)	Ready-to-Use (1.61µg/mL)	1:100 (7µg/mL)
Platform	Dako Autostainer Link 48, Agilent, Santa Clara, CA	BOND-MAX, Leica, Wetzlar, Germany	BOND-MAX, Leica, Wetzlar, Germany	Ventana BenchMark XT, Roche Ventana, Tuscon, AZ	Ventana BenchMark XT, Roche Ventana, Tuscon, AZ	BOND-MAX, Leica, Wetzlar, Germany
Compartment scored	TC membrane	TC membrane	TC membrane	TC and tumor-infiltrating IC	TC membrane	TC and tumor-infiltrating IC
Thresholds for positivePD-L1 expression	≥1%; ≥50% of TC	≥1% of TC	≥1% of TC	≥1% of TC; ≥50% of TC or ≥10% of tumor area with IC (if TC <50%)	1% [6]; ≥25% of TC [7]	≥1% of TC; ≥50% of TC or ≥10% of tumor area with IC (if TC <50%)

IC, immune cells; TC, tumor cell.

Supplementary Table 2: Sensitivity and specificity of respective IHC protocols to identifying PD-L1 RNA positive cases

IHC Protocol	Sensitivity	Specificity	*p*-value
22C3/Link48 TC1	5/6 (83%)	4/12 (25%)	0.615
22C3/Link48 TC50	1/6 (17%)	11/12 (92%)	1.000
28-8/BOND-MAX TC1	4/6 (67%)	10/12 (83%)	0.107
E1L3N/BOND-MAX TC1	6/6 (100%)	7/12 (58%)	0.038
SP142/BenchMark TC1	5/6 (83%)	7/12 (58%)	0.152
SP142/BenchMark TC50	1/6 (17%)	12/12 (100%)	0.333
SP263/BenchMark TC1	6/6 (100%)	3/12 (25%)	0.515
SP263/BenchMark TC25	4/6 (67%)	10/12 (83%)	0.107
SP142/BOND-MAX TC1	6/6 (100%)	0/12 (0%)	1.000
